# Effects of Hydrogen-Rich Water on Antioxidant Capacity, Immune Response, and Gut Microbiota in Juvenile Snakehead (*Channa argus*)

**DOI:** 10.3390/ani16132026

**Published:** 2026-07-02

**Authors:** Jiayi Wen, Junru Hu, Paini Xin, Songwei Chen, Huixiang Li, Yongchun Lin, Ying Yang, Yongsheng Wang

**Affiliations:** 1 School of Animal Science and Technology, Foshan University, Foshan 528225, China; wjy0896863@163.com (J.W.); 15994949802@163.com (P.X.); csw954086985@163.com (S.C.); lhxleee@163.com (H.L.); 2 Kunpeng Institute of Modern Agricultural Research at Foshan, Foshan 528225, China; 3 Institute of Animal Science, Guangdong Academy of Agricultural Sciences, Guangzhou 510640, China; hujunru1025@163.com; 4 Guangdong Cavolo Hydrogen Technology Company Ltd., Foshan 528200, China; 13929937817@163.com; 5 Shenzhen Branch, Guangdong Laboratory of Lingnan Modern Agriculture, Key Laboratory of Livestock and Poultry Multi-Omics of MARA, Agricultural Genomics Institute at Shenzhen, Chinese Academy of Agricultural Sciences, Shenzhen 518000, China

**Keywords:** hydrogen-rich water, antioxidant capacity, inflammatory response, gut microbiota, juvenile snakehead (*Channa argus*)

## Abstract

Hydrogen-rich water (HRW) has growth-promoting, antioxidant, anti-inflammatory, and microbiota-modulating properties; however, its effects on juvenile snakeheads (*Channa argus*) remain underexplored. In this experiment, we investigated the effects of HRW on the antioxidant capacity, immune response, and gut microbiota of juvenile snakeheads. A total of 360 fish (15.32 ± 0.50 g) were randomly assigned to three groups: a control group (aerated water), a low-hydrogen group (H1, 280 ± 50 ppb), and a high-hydrogen group (H2, 550 ± 50 ppb). The results revealed that, compared with the control group, H1 significantly reduced serum levels of total protein, triglycerides, glucose, and urea nitrogen (*P* < 0.05). In the liver, H1 upregulated *tir-2* expression and downregulated *tnf-α* expression (*P* < 0.05). Furthermore, serum SOD activity was significantly increased in both H1 and H2 groups compared with the control (*P* < 0.05), while serum CAT activity was significantly elevated only in the H2 group (*P* < 0.05). No significant alteration was detected in intestinal morphology (*P* > 0.05), and similarly, digestive enzyme activity did not change significantly (*P* > 0.05). Regarding gut microbiota, the H1 group increased the relative abundance of *Actinobacteria* (*P* < 0.05); at the genus level, it decreased the abundance of *Acinetobacter* and *Pseudomonas*, while increasing the abundance of *Agathobacter*, *Faecalibacterium*, and *Anaerostipes* (*P* < 0.05). In summary, our findings revealed that optimal HRW supplementation enhances immunomodulatory and antioxidant functions and improves intestinal health, thereby establishing HRW as a promising functional water conditioner for sustainable aquaculture applications.

## Simple Summary

Hydrogen-rich water has antioxidant and anti-inflammatory properties that may benefit farmed fish health, but this has not been confirmed in juvenile snakehead. In this study, hydrogen-rich water at three different concentrations (0 ppb, 280 ± 50 ppb, and 550 ± 50 ppb) was added to the rearing water of juvenile snakehead for 56 days. Growth performance, blood biochemical parameters, antioxidant enzyme activities, immune-related gene expression, intestinal structure, digestive enzyme activities, and gut bacteria composition were measured. The results showed that the low concentration H1 group (280 ± 50 ppb) reduced blood stress markers, lowered the expression of an inflammation-related gene, increased antioxidant enzyme activities, and optimized the gut microbiota composition. Therefore, adding low-concentration hydrogen-rich water to rearing water is a simple and safe method to improve immune function, antioxidant defense, and gut health in juvenile snakehead, thereby supporting more sustainable aquaculture.

## 1. Introduction

Juvenile snakehead (*Channa argus*) is a freshwater aquaculture species with significant economic value and is renowned for its delicious meat and rich nutritional content. It has emerged as a significant source of aquatic protein [1]. With continuously growing market demand, the scale of its farming has expanded steadily, solidifying its industrial position [2]. However, issues such as germplasm degradation and deteriorating farming environments have become increasingly severe, constraining the sustainable development of this industry [3]. Therefore, the development of healthy and efficient aquaculture regulation technologies to improve the physiological status of fish has become a critical demand for industrial advancements.

Molecular hydrogen (H_2_), having a unique antioxidant activity, selectively neutralizes harmful reactive oxygen species, such as hydroxyl radicals, without interfering with normal redox signaling [4]. Its role in alleviating oxidative stress in mammals and plants is well-documented [5,6,7]. Hydrogen-rich water (HRW), as a carrier of H2, not only retains the biological activity of hydrogen but also exhibits anti-inflammatory, anti-apoptotic, and gene expression regulatory effects [8,9], demonstrating promising applications in enhancing metabolic activity and stress resistance in agriculture and medicine [10,11]. In practical aquaculture, HRW requires specialized equipment (e.g., hydrogen generators and on-line monitors) compared to traditional antioxidant additives such as vitamin C or vitamin E. However, it offers advantages including no chemical residues, low running costs (only water and electricity), and high safety, making it a viable alternative for sustainable fish farming.

Aquatic animals under intensive farming systems are prone to oxidative stress, which leads to growth inhibition and immune dysregulation [12]. The past decade has witnessed emerging reports on HRW applications in aquaculture, such as enhancing antibacterial immunity in zebrafish [13] and mitigating oxidative damage in rainbow trout [14]. The intestine is a key site of digestion, immunity, and microbial regulation in fish. The stability of its structure and microbiota is crucial for maintaining host health [15,16]. Preliminary studies have indicated that HRW can modulate the composition of fish gut microbiota [17,18]. Nevertheless, systematic investigations of the combined effects of HRW on antioxidant function, inflammatory responses, and gut microbiota in juvenile snakeheads are still lacking. Specifically, the concentration-dependent effects of HRW on these parameters in this species have not been elucidated. Based on the above, we propose the following hypothesis: HRW supplementation can alleviate rearing-related stress in juvenile snakeheads through a multi-faceted mechanism involving enhanced antioxidant capacity, suppression of inflammation, and modulation of the gut microbiota, and that these effects are concentration-dependent.

Therefore, in this study, we systematically evaluated the effects of adding different concentrations of HRW to aquaculture water on the antioxidant status, inflammatory markers, and gut microbiota structure of juvenile snakeheads. Herein, we aimed to provide a theoretical basis for the rational application of HRW in the healthy farming of this fish species, thereby promoting the ecologically sound and sustainable development of aquaculture.

## 2. Materials and Methods

### 2.1. Fish and Feeding Management

Juvenile snakeheads were purchased from Jinyuan Agricultural and Animal Husbandry Products Development Co., Ltd. (Yingde, China). The experiment was conducted in an indoor recirculating aquaculture system at the Kunpeng Institute of Modern Agricultural Research in Foshan. Before the experiment, the fish were temporarily reared for 7 days. During the experiment, they were fed acommercial diet (crude protein > 48.20%, crude lipid > 11.30%, crude ash < 12.60%; Foshan Jieda Feed Co., Ltd., Foshan, China). A total of 360 healthy juvenile fish with uniform body weight (15.32 + 0.50 g) were randomly divided into three groups. The experiment was carried out in three aquaculture tanks, each measuring 4.6 m in length, 4 m in width, and with a water depth of 33 cm. Four net cages (60 cm × 60 cm × 40 cm, length × width × height) were placed in each tank (with 4 replicates per group and 30 fish per replicate), and the experiment lasted 56 days. The net cages were submerged so that their bottoms were underwater, and the tops extended approximately 7 cm above the water surface to prevent fish from escaping. The three treatment groups were: a control group without hydrogen supplementation (0 ppb) and two experimental groups receiving 10 h of daily hydrogen supplementation, H1 (280 ± 50 ppb) and H2 (550 ± 50 ppb), respectively. Hydrogen generation and monitoring equipment (model HIM-19-07) was provided by Guangdong Cavolo Hydrogen Technology Co., Ltd. (Foshan, China). Hydrogen was generated by water electrolysis using a PE proton exchange membrane. To maintain and verify the actual hydrogen concentration during the rearing period, the H2 concentration in each tank was measured twice daily (at 08:00 and 16:00) using a portable dissolved hydrogen meter (ENH 2000, TRUSTLEX, Tokyo, Japan). The water in each tank was completely renewed every 24 h, and fresh hydrogen-rich water was prepared immediately before each water change to maintain the intended hydrogen levels. Throughout the experimental period, the fish received feed twice daily at 09:00 and 18:00. The daily feeding rate was adjusted according to the feed intake of the previous day, ranging from 3% to 5% of the total bodyweight of juvenile snakeheads in each tank. The water temperature was kept at 25.0 ± 1.0 °C throughout the experimental period, while the pH was 8.00 ± 0.50 and the dissolved oxygen level was 6.0 ± 1.0 mg/L.

### 2.2. Sample Collection

At the end of the experiment, the juvenile snakeheads were fasted for 24 h and then weighted and counted. Four fish were randomly selected from each treatment group (12 fish in total) for dissection, and liver and blood samples were collected. A total of 6 fish per group were randomly selected for intestinal analysis. To obtain sufficient tissue and minimize individual variation, every two intestinal samples were pooled into one biological replicate, resulting in three replicates per treatment group (*n* = 3). To prepare a 10% tissue homogenate, 0.10 g of liver tissue was mixed with 0.9 mL of 0.9% saline and homogenized in an ice–water bath at 4.0 °C. Moreover, whole fish specimens were used for routine nutritional analyses. Subsequently, the blood samples were centrifuged at 4.0 °C and 1200× *g* for 10 min to separate the serum, which was then aliquoted and stored at −80 °C. Liver samples were stored at −80 °C for subsequent RNA extraction. Intestinal tissues were preserved in 4% paraformaldehyde solution and stored in 10 mL centrifuge tubes for histological analysis. Finally, intestinal contents were stored at −80 °C for DNA extraction, which were used for intestinal microbiota analysis.

### 2.3. Growth Performance and Whole-Body Proximate Composition Analysis

The weight gain rate (WGR), specific growth rate (SGR), feed conversion ratio (FCR), feed intake rate (FR), and survival rate (SR) of juvenile snakehead were calculated using Equations (1)–(5) as follows:

(1) $$SR\;\left(\%\right)=\;\left.\left(Final\;fish\;count/Initial\;fish\;count\right.\right)\times 100\%$$

(2) $$WGR\;\left(\%\right)=\left.[(Final\;body\;weight-Initial\;body\;weight)/Initial\;body\;weight\right]\times 100\%$$

(3) SGR (%/day)=[ (ln Final  weight−ln Initial  weight)/Feeding days]×100%

(4) $$FCR\;=\;Total\;feed\;intake/\left(Final\;total\;weight-Initial\;total\;weight\right)$$

(5) FR (g/fish)=100×Total feed intake / [Culture days×(Final total weight+Initial total weight)]

To assess the moisture, crude protein, crude lipid, crude ash, calcium (Ca), and phosphorus (P) contents of the whole fish body, proximate analyses were conducted following standard procedures [19]. Moisture levels were tested by drying the sample to constant weight in an oven set at 105 °C. Ash content was analyzed using a muffle furnace set at 550 °C. Crude protein was quantified using the Kjeldahl method with an automated analyzer (Kjeltec 2300; FOSS Tecator, Höganäs, Sweden), and crude lipid content was assessed using Soxhlet extraction (HT6 System; Tecator, Höganäs, Sweden). Ca content was analyzed by EDTA complexometric titration using calcon carboxylic acid as an indicator. P content was analyzed by the molybdenum blue colorimetric method, and the absorbance of the phosphomolybdenum blue complex was measured at 880 nm. Crude protein, crude fat, ash, calcium, and phosphorus contents were expressed on a wet weight basis by correcting the dry weight values with the corresponding moisture content of each sample.

### 2.4. Analysis of Serum Biochemical and Antioxidant Parameters

Serum samples from individual fish (*n* = 4 per group) were analyzed using commercial kits (Nanjing Jiancheng Biological Engineering Institute, Nanjing, China). Serum biochemical parameters, including the levels of blood urea nitrogen (BUN), glucose (GLU), total cholesterol (TC), triglycerides (TGs), high-density lipoprotein cholesterol (HDL-C), and low-density lipoprotein cholesterol (LDL-C), were quantitatively measured using a fully automated biochemical analyzer (Hitachi 7600, Tokyo, Japan). To evaluate serum antioxidant capacity, the activities of superoxide dismutase (SOD), catalase (CAT), and glutathione peroxidase (GSH-Px), as well as the levels of total protein (TP), hydrogen peroxide (H_2_O_2_), malondialdehyde (MDA), and nitric oxide (NO), were determined using corresponding commercial kits (Nanjing Jiancheng Bioengineering Institute Co., Ltd., Nanjing, China) following the manufacturer’s instructions.

### 2.5. Real-Time Quantitative Polymerase Chain Reaction (qPCR)

Total RNA was extracted from snakehead livers of each group (*n* = 4 per group, with each fish originating from an independent tank; total 12 fish across three groups) using the Eastern^®^ Super Assay Kit (Promega, Shanghai, China). RNA quality and integrity were detected using a Quawell Q5000 Ultra-Micro Nanodrop spectrophotometer (Quawell Technology, San Jose, CA, USA; OD 260/280 values in the range of 1.80–2.10; OD 260/230 values in the range of 1.90–2.10), and extracted RNA was electrophoresed on a 1% agarose gel. We used the Golddenstar™ RT6 cDNA Synthesis kit (Tsingke, Beijing, China) to generate cDNA, which was then stored at −20 °C. qPCR was conducted on a QuantStudio 5 system (Applied Biosystems, Foster City, CA, USA) with Perfect Start^®^ Green qPCR SuperMix (TransGen Biotech, Beijing, China). The cycling conditions were: 94 °C for 30 s (initial denaturation); 45 cycles of 94 °C for 5 s and 60 °C for 30 s; followed by a melting curve from 60 °C to 95 °C. The reference gene was β-actin. Primer design was performed with Primer 6 (Table 1), and the primers were synthesized by TSINGKE Biological Technology Co., Ltd. (Guangzhou, China). The relative expression of target genes was calculated using the 2^−ΔΔCt^ method.

### 2.6. Intestinal Histology Analyses

The fixed intestinal tissues were immersed in a 4% paraformaldehyde solution, sliced into routine paraffin sections, with a section thickness of 6.0 μm, and stained with hematoxylin–eosin. We scanned and photographed the sections with a panoramic digital scanner (PANNORAMICDESK/MIDI/250/1000; 3DHISTECH, Budapest, Hungary). The resulting tissue section images were captured using CaseViewerTM 2.4 (3DHISTECH). After image acquisition, the whole-slide images of each intestinal sample were imported into Image-Pro Plus 6.0 (Media Cybernetics, Inc., Rockville, MD, USA) analysis software. For each of the four samples per group, one representative image was selected, and the following parameters were measured at 20× magnification: villus height (vertical distance from the villus tip to the crypt-villus junction), villus width (measured at the villus midpoint), muscularis propria thickness (vertical distance from the inner circular muscle layer to the outer longitudinal muscle layer), and mucosal propria thickness (vertical distance from the basal side of the intestinal epithelium to the muscularis mucosae). The scale was calibrated using a stage micrometer prior to measurement. A total of four measurements (one per sample) were obtained for each group.

### 2.7. Analysis of Intestinal Digestive Enzyme Activities

Intestinal digestive function was evaluated by determining the activities of amylase (AMS), trypsin (TRY), and lipase (LPS) in intestinal tissue homogenates using commercial assay kits (Nanjing Jiancheng Bioengineering Institute, Nanjing, China).

### 2.8. Intestinal Microbiota Analyses

The genomic DNA of gut microbiota was extracted from the gut contents of juvenile snakeheads using the FastDNA^®^ Spin Kit for Soil (MP Biomedicals, Solon, OH, USA). All samples were sequenced and analyzed by Majorbio Bio-Pharm Technology Co., Ltd. (Shanghai, China). Amplification of the V3–V4 hypervariable region of the 16S rRNA gene was performed using primers 338F (5′-ACTCCTACGGGAGGCAGCAG-3′) and 806R (5′-GGACTACHVGGGTWTCTAAT-3′). The amplicons were verified by 2% agarose gel electrophoresis and purified using the AxyPrep DNA Gel Extraction Kit (Axygen, Union City, CA, USA). Sequencing libraries were created using the TruSeq^®^ DNA PCR-free Sample Preparation kit (Illumina, San Diego, CA, USA), and each sample was indexed with a unique barcode. The libraries were subjected to 2 × 300 bp paired-end sequencing on the Illumina MiSeq platform (Illumina, San Diego, CA, USA). The raw sequences have been deposited in the GenBank database (Accession No. PRJNA1235928). Using fastp software (version 0.20.0), we filtered the obtained paired-end reads for quality with a 50 bp sliding window and a minimum average quality score of 20. After trimming, the reads were assembled with FLASH software (version 1.2.7) using default parameters. The merged sequences were clustered into operational taxonomic units (OTUs) at 97% similarity threshold using UPARSE software (version 7.0.1090), with chimeric sequences removed during clustering. Each OTU representative sequence was taxonomically classified using the RDP classifier (version 11.5) against the Silva 16S rRNA database (release 138), with a confidence threshold of 0.7. Alpha diversity indices, including Sobs, Shannon, Simpson, Ace, and Chao 1, were calculated on the Majorbio Cloud Platform. Based on Bray–Curtis distances, beta diversity was evaluated via Principal Component Analysis (PCA), Principal Co-ordinates Analysis (PCoA), and Non-metric Multidimensional Scaling (NMDS). Additionally, to assess differences in overall microbial composition among groups, partial least squares discriminant analysis (PLS-DA) was performed on the same platform.

### 2.9. Statistical Analyses

All data were first assessed for normality using the Shapiro–Wilk test and for homogeneity of variances using Levene’s test. After the normality and homoscedasticity assumptions were confirmed, one-way ANOVA was performed to compare group differences, with Duncan’s multiple range test used for post hoc comparisons. For data that did not meet the assumptions of normality or homogeneity of variances, the Kruskal–Wallis H test was used as a non-parametric alternative. Results are presented as mean pooled SEM (standard error of the mean). Statistical significance was set at *P* < 0.05. All statistical analyses were performed with SPSS version 20.0 (SPSS Inc., Chicago, IL, USA).

## 3. Results

### 3.1. Effects of HRW on Growth Performance

The analysis results for the effects of HRW supplementation on the growth performance and whole-body composition of snakeheads over an 8-week experimental period are presented in Table 2. No significant differences were observed between the HRW-treated groups (H1 and H2) and the control group in terms of SR, FR, FCR, SGR, IBW, WGR, or FW (*P* > 0.05). However, the low-HRW group (H1) had significantly higher WGR and FW than the high-HRW group (H2) (*P* < 0.05).

### 3.2. Effects of HRW on Whole-Body Composition

The whole-body proximate composition (crude protein, crude lipid, crude ash, moisture, calcium, and total phosphorus) showed no significant variation among the groups (*P* > 0.05), as shown in Table 3.

### 3.3. Effects of HRW on the Serum Biochemistry of Juvenile Snakehead

As shown in Table 4, exposure to H2 decreased the serum levels of TP, TGs, GLU, and BUN, with the H1 group exhibiting significantly lower levels than those in the control group (*P* < 0.05). The levels of HDL-C, TC, and LDL-C in the hydrogen treatment groups were not significantly different from those in the control group (*P* > 0.05).

### 3.4. Effects of HRW on Serum Antioxidant Capacity

**Figure 1:**
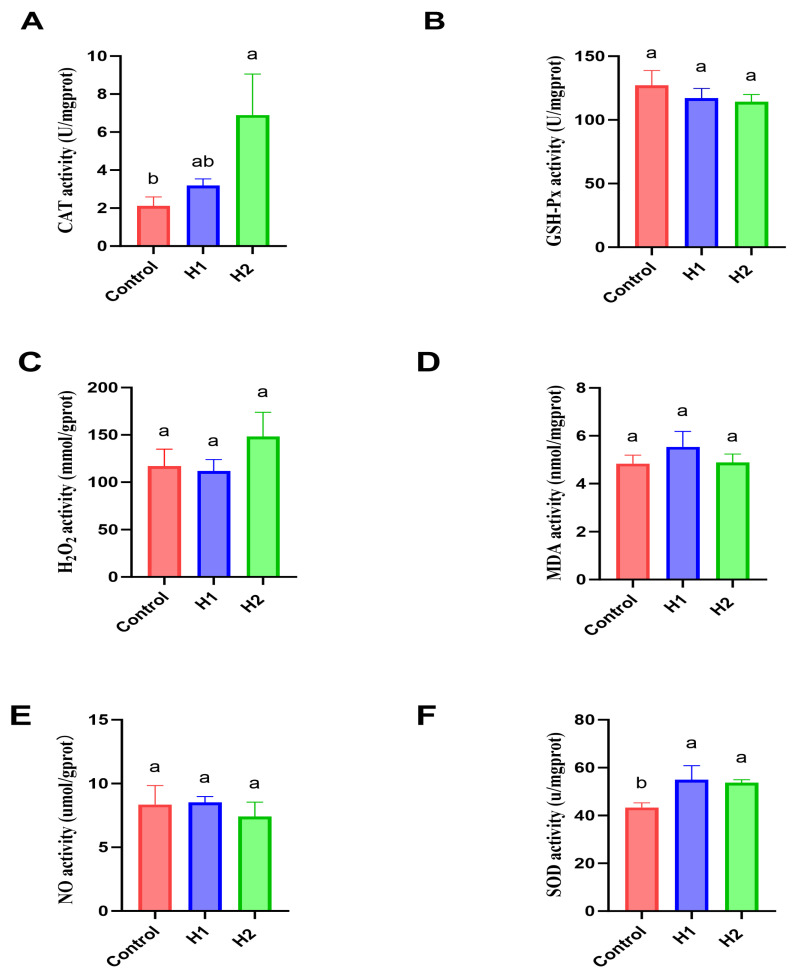
Effects of HRW on serum antioxidant capacity in juvenile snakehead. (**A**) Catalase activity (CAT); (**B**) Glutathione peroxidase activity (GSH-Px); (**C**) Hydrogen peroxide content (H_2_O_2_); (**D**) Malondialdehyde content (MDA); (**E**) Nitric oxide content (NO); (**F**) Superoxide dismutase activity (SOD). Values are means + S.E. (*n* = 4). Different lowercase letters above the bar represent significant differences between groups (*P* < 0.05).

Compared with the control, CAT activity was significantly elevated in the high-concentration hydrogen group (H2) (*P* < 0.05). SOD activity was also increased significantly in all hydrogen-treated groups (*P* < 0.05). By contrast, no significant differences were observed in the serum levels of GSH-Px, H_2_O_2_, MDA, or NO between the treatment groups and the control group (Figure 1, *P* > 0.05).

### 3.5. Effects of HRW on Hepatic Gene Expression

**Figure 2:**
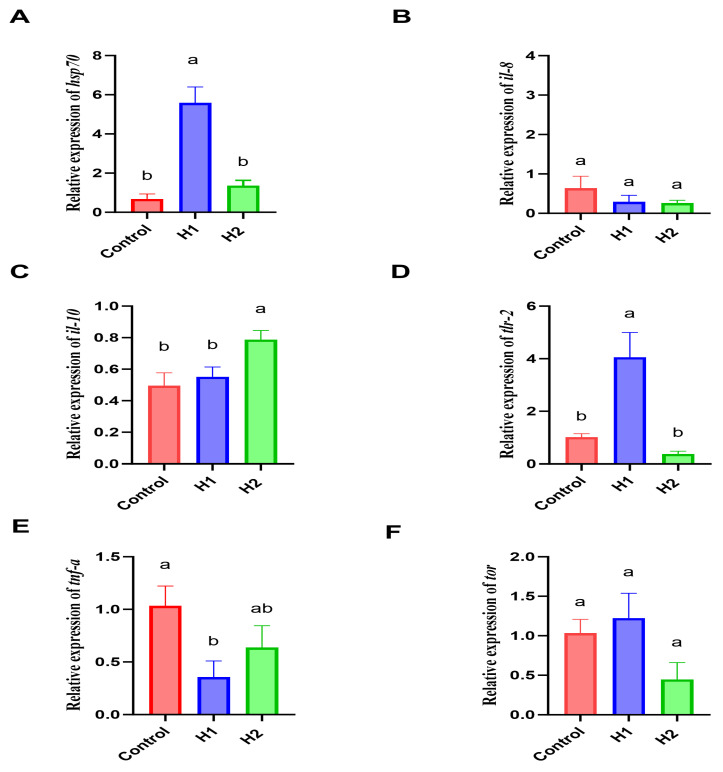
Effect of HRW on gene expression in the liver of juvenile snakehead. (**A**) Heat shock protein 70 (*hsp70*); (**B**) Interleukin-8 (*il-8*); (**C**) Interleukin-10 (*il-10*); (**D**) Toll-like receptor 2 (*tlr-2*); (**E**) Tumor necrosis factor-α (*tnf-α*); (**F**) Target of rapamycin (*tor*). Values are means + S.E. (*n* = 4). Different lowercase letters above the bar represent significant differences between groups (*P* < 0.05).

Furthermore, HRW supplementation significantly enhanced the relative expression of *tlr-2* and *hsp70* in the H1 (*P* < 0.05) while reducing the expression of *tnf-a*. The H2 group exhibited enhanced expression of *il-10* (*P* < 0.05). However, the HRW treatment did not significantly affect the expression levels of *tor* and *il-8* (Figure 2, *P* > 0.05).

### 3.6. Histological Observations

**Figure 3:**
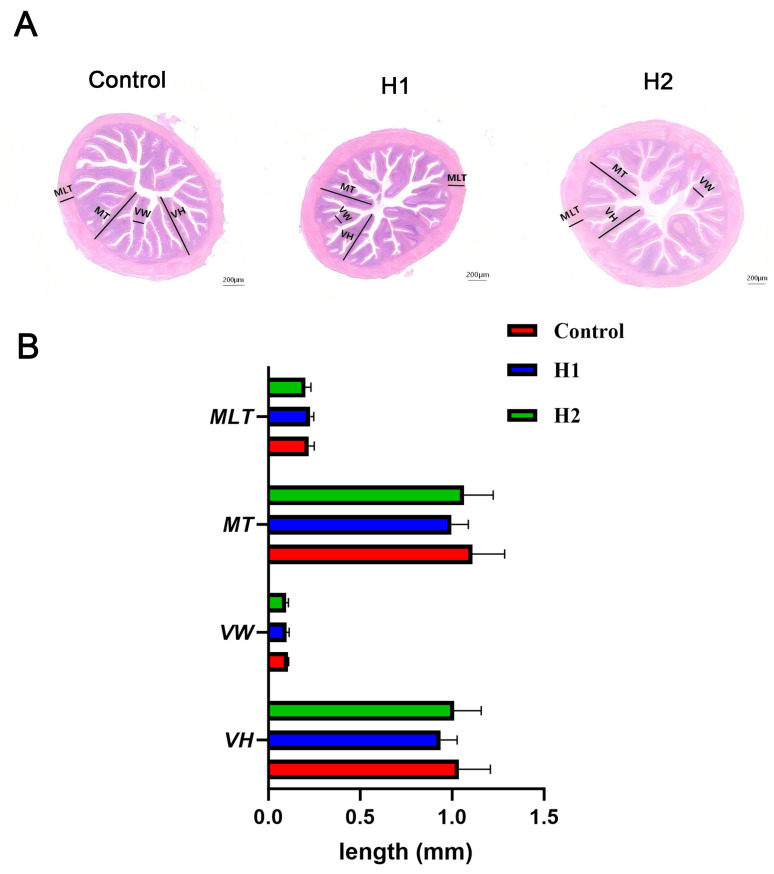
Effects of HRW supplementation on the intestinal histology of juvenile snakehead. (**A**) Intestinal histological changes; (**B**) Quantitative analysis of intestinal morphology. Values are means + S.E. (*n* = 4). MLT: Muscle layer thickness; MT: Mucosal thickness; VW: Villus width; VH: Villus height.

In this study, hydrogen administration did not induce significant structural alterations in the intestinal tissues of snakeheads (Figure 3A). Furthermore, no statistically significant differences were observed in key morphological measurements (MLT, MT, VW, and VH) when comparing the hydrogen-treated groups (H1 and H2) with the control group, according to quantitative evaluations (Figure 3B, *P* > 0.05).

### 3.7. Effects of HRW on Intestinal Digestive Enzymes

**Figure 4:**
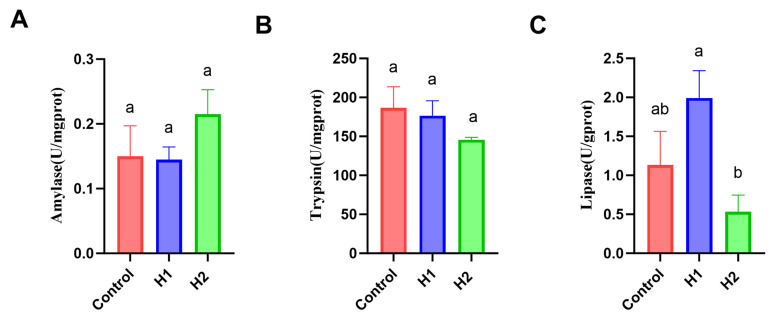
Effect of HRW on intestinal enzyme activities in juvenile snakehead. (**A**) Amylase activity; (**B**) Trypsin activity; (**C**) Lipase activity. Values are means + S.E. (*n* = 4). Different lowercase letters above the bar represent significant differences between groups (*P* < 0.05).

The results revealed that HRW dosing did not affect the activities of intestinal enzymes, such as AMS, TRY, and LPS, in any of the groups, and the changes were not significant when compared with those in the control group (Figure 4, *P* > 0.05).

### 3.8. Effects of HRW on Intestinal Microbial α-Diversity

Alpha diversity of the intestinal microbiota is shown in Table 5. No significant intergroup differences were detected in the Ace, Chao 1, Shannon and Sobs diversity indices (*P* > 0.05). In particular, Simpson’s index exhibited a gradual increase with increasing hydrogen concentrations, with the H2 group exhibiting a significant increase compared with that in the control group (*P* < 0.05).

### 3.9. HRW Regulates the Intestinal Microbial Community

**Figure 5:**
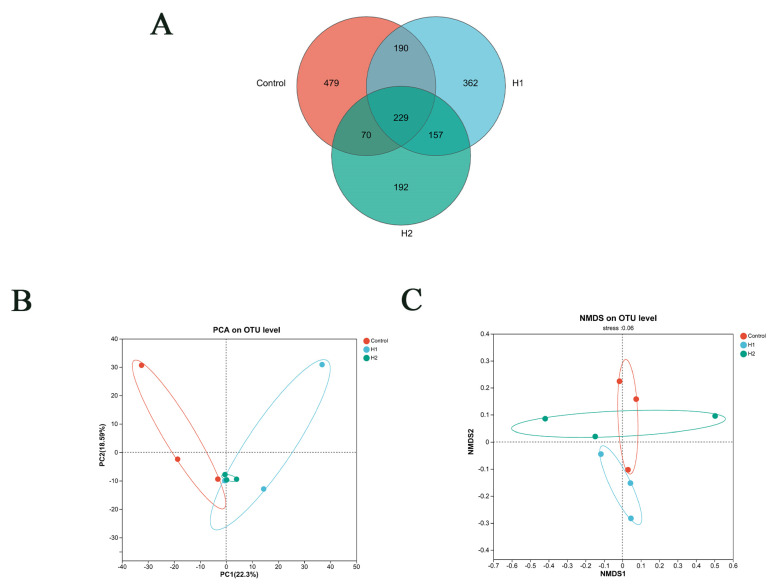
(**A**) Effect of HRW on OTU levels in the gut microbiota of three groups (**B**) Principal component analysis for each group (**C**) Non—metric multidimensional scaling analysis of each.

The rRNA sequencing of fecal samples from the gut flora of different juvenile snakeheads was performed using 16S rRNA sequencing. The operational taxonomic units shared by the control, H1, and H2 groups were visualized using Venn diagrams (Figure 5A). Principal component analysis and nonmetric multidimensional scaling revealed that HRW supplementation altered the overall microbial composition compared with that in the control group, suggesting that HRW may induce changes in gut microbiome profile (Figure 5B,C).

**Figure 6:**
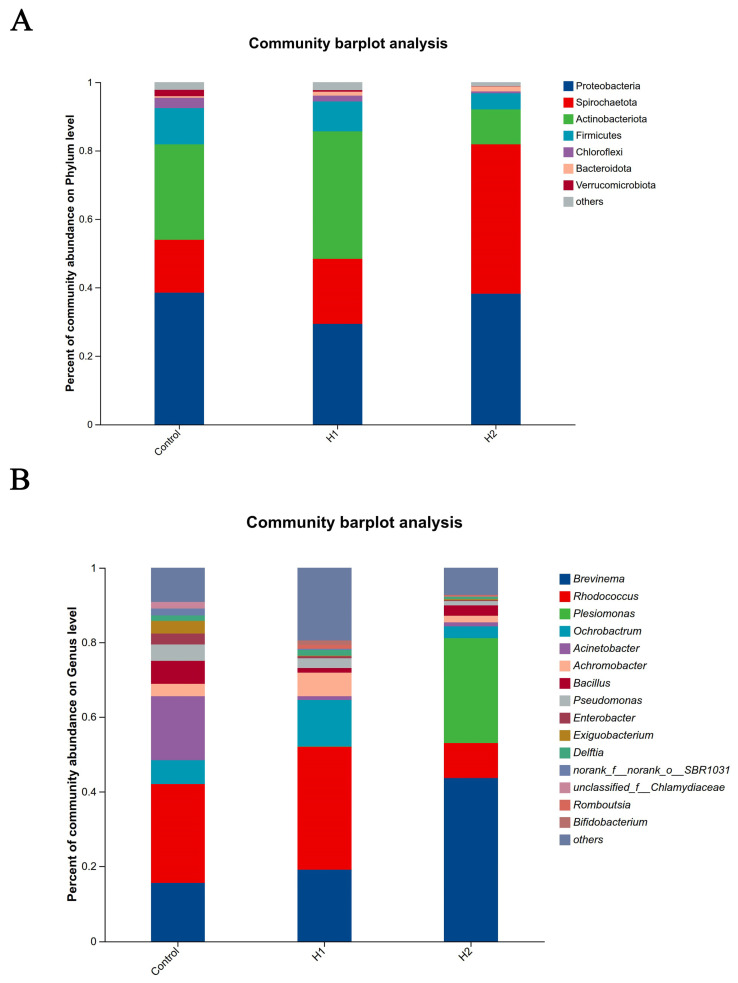
The relative abundance of intestine microbiota composition in juvenile snakehead at the phylum level (**A**) and genus level (**B**).

At the phylum level, compared to the control group, the relative abundance of *Actinobacteria* increased in the H1 group, whereas that of *Proteobacteria* and *Firmicutes* decreased. In contrast, in the H2 high-concentration hydrogen group, the relative abundance of *Spirochaetes* increased, whereas those of *Firmicutes* and *Actinobacteria* decreased (Figure 6A). At the genus level, the H1 group exhibited increased relative abundances of *Rhodococcus*, *Achromobacter*, *Ochrobactrum*, and *Norank_f_norank_o_SBR1031*, whereas those of *Pseudomonas*, *Bacillus*, *Enterobacter*, and *Acinetobacter* decreased. In comparison, the H2 group exhibited increased relative abundances of *Brevinema* and *Plesiomonas* and decreased abundances of *Acinetobacter*, *Achromobacter*, *Rhodococcus*, and *Pseudomonas* (Figure 6B).

**Figure 7:**
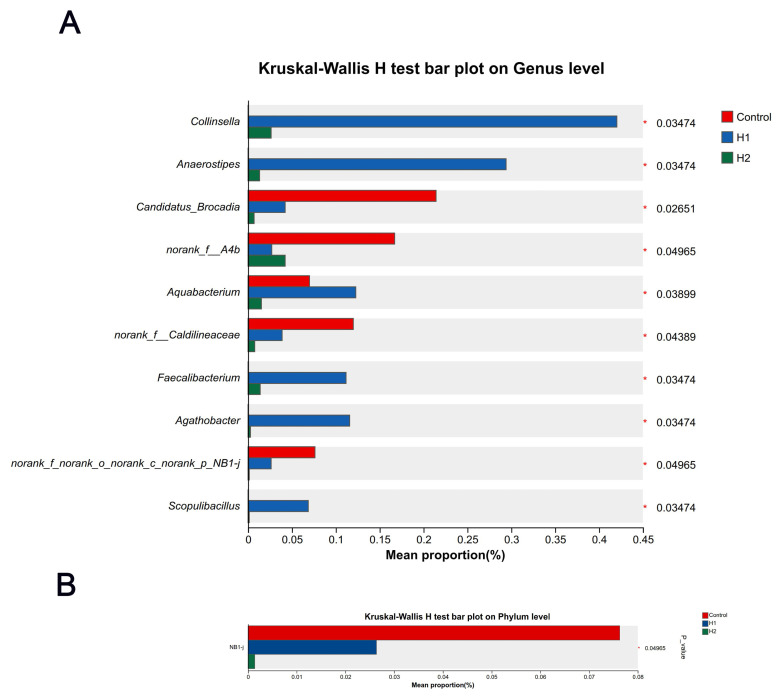
Effects of HRW on differences in gut microbiota composition in juvenile snakehead at (**A**) genus level and (**B**) phylum level. The asterisk (*) indicates that the relative abundance of the corresponding taxon differed significantly among groups according to the Kruskal–Wallis H test (*P*< 0.05).

Comparative analysis of the community structure identified significant differences at the genus level between the H1 treatment and control groups. The H1 group exhibited significantly higher relative abundances of *Collinsella*, *Anaerostipes*, *Aquabacterium*, *Faecalibacterium*, *Agathobacter*, and *Scopulibacillus* than the control group *(P* < 0.05, Figure 7A). Conversely, significantly lower abundances were detected for *Candidatus_Brocadia*, *norank_f_A4b*, *norank_f_Caldilineaceae*, and *norank_f_norank_o_norank_c_n* in the H1 group (*P* < 0.05, Figure 7A). At the phylum level, the H1 group exhibited a significantly decreased relative abundance of *NB1j* compared to controls (*P* < 0.05, Figure 7B).

**Figure 8:**
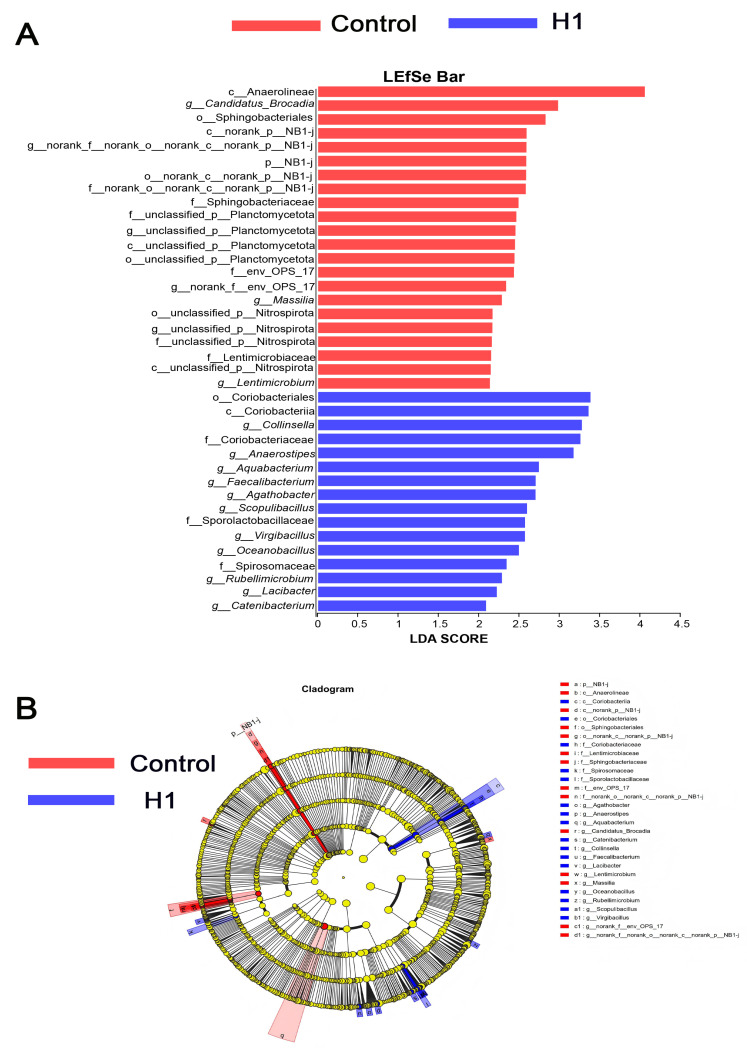
Analysis of linear discriminant analysis (LDA) effect size (LEfSe) of HRW on the gut microbiota of juvenile snakehead. (**A**) Histogram of the distribution of intestinal LDA values of juvenile snakehead in each group (less strict is set as 2; more strict is set as 3). (**B**) Branching diagram of gut microbial evolution in juvenile snakehead of each group.

To analyze HRW’s impact on intestinal microbes of juvenile snakeheads, we performed LEfSe (linear discriminant analysis effect size). Species with LDA scores > 3 were identified from the three sample groups (Figure 8A,B). The results showed that the differences between the bacteria were statistically significant in all groups, except for the H2 group (*P* < 0.05). In LDA discrimination, the H1 hydrogen concentration group showed a significant (*P* < 0.05) increase in the relative abundance of *o__Coriobact eriales*, *c__Coriobacteriia*, *g__Collinsella*, *f__Coriobacteriaceae*, and *g__Anaerostipes* compared with the control group. In contrast, the H1 group exhibited significantly lower *c_Anaerolineae* relative abundance than controls (*P* < 0.05).

## 4. Discussion

### 4.1. Effects of HRW on Growth Performance and Physiological Homeostasis in Juvenile Snakehead

The results of the present study indicate that the effect of HRW on the growth of juvenile snakeheads is concentration-dependent. Specifically, compared with the H2 group, the H1 group had significantly higher final weights and WGR. In contrast, none of the treatment groups differed significantly from the control group. Additionally, no significant changes were detected in the basic nutritional components of juvenile snakehead. This concentration-dependent, limited growth-promoting effect contrasts with the pronounced growth enhancement observed in largemouth bass [20] and aligns with common reports in mammalian models showing no direct growth-promoting effects [21]. The growth advantage of H1 over H2 indicates the critical importance of the optimal dosage because lower concentrations may promote growth by optimizing metabolism and alleviating stress. Furthermore, the 8-week intervention period may have been relatively brief, potentially contributing to the lack of significant growth differences between the treatment and control groups. Thus, the growth-promoting effect of HRW on juvenile snakeheads manifests as a concentration-dependent physiological regulation and could be modulated by factors such as treatment duration. Moreover, the 8-week treatment period might be insufficient to reveal significant differences in healthy, rapidly growing juveniles. Future studies should extend the intervention time or use stressed/infectious models to better assess growth-promoting potential.

### 4.2. Effects of HRW on Serum Biochemical Parameters and Antioxidant Capacity in Juvenile Snakehead

Serum physiological and biochemical parameters are crucial indicators for evaluating the growth, metabolism, and overall health of fish [22]. TP, TGs, GLU, and BUN are important markers of lipid and protein metabolism. In the present study, H1 significantly reduced the serum levels of GLU, TG, and BUN in snakeheads, with H1 showing better efficacy than H2, which may be attributed to the concentration-dependent effects of HRW. These changes suggest that low-dose HRW may enhance the overall metabolic efficiency of snakeheads by synergistically optimizing the carbohydrate, lipid, and protein metabolic pathways. Similar metabolic improvements have been reported with other functional feed additives [23]. For instance, Ramulus mori oligosaccharides have been shown to significantly reduce serum TG and TC levels, while increasing SOD activity, thereby promoting lipid metabolism in largemouth bass [24]. In terms of antioxidant capacity, SOD and CAT are key enzymes in the antioxidant defense system of fish, playing vital roles in scavenging superoxide radicals and maintaining redox balance [25,26]. The present study found that HRW treatment significantly enhanced SOD activity in both H1 and H2 and increased CAT activity only in the H2, which aligns with the established antioxidant-enhancing properties of HRW [27]. These findings are consistent with those of previous studies in various biological systems [28,29]. This differential response suggests that CAT may require a higher H_2_ concentration to be upregulated, possibly because CAT gene expression is less sensitive to low H_2_ levels compared to SOD.

### 4.3. HRW Regulates the Expression of Inflammatory Factors in the Liver of Juvenile Snakehead

The hepatic immune response in fish involves coordinated interactions between immunologically active cells and cytokines, including ILs and *tnf-α*, which are the key mediators of inflammatory regulation [30]. Keeping pro-inflammatory and anti-inflammatory cytokines in balance is essential to tissue integrity and immune homeostasis [1]. The results obtained in the present study revealed that HRW intervention differentially regulated the expression of immune-related genes. The H2 exhibited a significant upregulation of the anti-inflammatory cytokine gene *il-10*, whereas the H1 exhibited an enhanced expression of *tlr-2* and significantly downregulated expression of the pro-inflammatory cytokine gene *tnf-α*. The elevated expression of *il-10*, a key anti-inflammatory mediator, effectively suppresses the synthesis and release of pro-inflammatory factors, thereby mitigating inflammatory cascades. The observed downregulation of *tnf-α* further corroborates the anti-inflammatory effects of the HRW treatments. These findings are consistent with those of mechanistic studies using various animal models. For example, HRW has been reported to reduce *tnf-α* but upregulate *il-10* expression through its antioxidant, anti-apoptotic, and anti-inflammatory properties, thereby improving recovery after ischemia–reperfusion injury in mice [31]. In experimental colitis, HRW mitigates tissue damage, enhances antioxidant capacity, and decreases *tnf-α* levels [32]. Similarly, HRW has been reported to suppress ethanol-induced increases in blood lipid levels and *tnf-α* expression while promoting *il-10* expression in a chronic liver injury model [33]. Therefore, the enhancement of immune capacity in snakehead may be attributed to changes in the expression levels of the *il-10* and *tnf-α* genes. Importantly, the opposing concentration-dependent effects (H1 mainly suppressing *tnf-α*; H2 mainly elevating *il-10*) indicate that the anti-inflammatory action of HRW may involve multiple pathways that are activated at different doses. This complexity should be considered when selecting HRW concentrations for immunomodulatory purposes.

### 4.4. HRW Regulates Intestinal Health in Juvenile Snakehead

Intestinal health is fundamental to the physiological functions of fish, with structural integrity and microbial homeostasis together supporting efficient nutrient absorption and immune competence [34,35]. HRW has been shown to improve intestinal morphology in terrestrial animals [36]. However, no significant structural changes were observed in juvenile snakeheads in the present study, which is consistent with previous reports on largemouth bass [37], and likely reflects species-specific responses in aquatic organisms or the capacity of healthy fish to maintain morphological homeostasis. However, the absence of structural changes does not preclude functional improvements, as evidenced by the substantial microbiota shifts.

Notably, the regulatory effects of the H1 on gut microbiota were particularly evident. In this group, the relative abundances of *Collinsella*, *Faecalibacterium*, *Anaerostipes*, *Agathobacter*, and *Scopulibacillus* increased significantly, suggesting that low-dose HRW preferentially supported the colonization and functional expression of taxa involved in metabolic regulation. These genera play key roles in host metabolic health. *Collinsella* modulates glucose and lipid metabolism by influencing cholesterol absorption and hepatic gluconeogenesis [38], *Faecalibacterium* strengthens the intestinal barrier through upregulation of tight-junction proteins [39], and *Anaerostipes* converts dietary inositol into short-chain fatty acids (SCFAs), contributing to glucose homeostasis [40]. SCFAs derived from the microbial fermentation of dietary fiber in the gut [41] are generally associated with improved intestinal health and enhanced glucose and lipid metabolism [42,43]. The enrichment of these SCFA-producing bacteria in the H1 group likely reflected greater SCFA production, which may, in turn, support more efficient glucose utilization and energy metabolism.

Moreover, the H1 group showed increased microbial alpha diversity, reduced abundance of *Pseudomonas* and *Acinetobacter*, and elevated levels of *Agathobacter*, *Anaerostipes*, and *Faecalibacterium*. Together, these shifts help strengthen intestinal barrier function and improve overall health resilience [44]. Importantly, the beneficial modifications in the gut microbiota observed in the H1 group corresponded to improvements in serum metabolic markers, e.g., decreased GLU, TG, and BUN, and antioxidant capacity (elevated SOD activity). These results indicate that low-concentration hydrogen-rich water may improve the physiological status of juvenile snakeheads through multi-dimensional synergistic regulation of antioxidant, metabolic, and microbiota pathways, thereby exerting more integrated and pronounced physiological effects within the experimental concentration range.

## 5. Conclusions

In summary, this study demonstrated that HRW supplementation exerts concentration-dependent beneficial effects on juvenile snakeheads (*Channa argus*). The low-concentration HRW (280 ± 50 ppb, H1) significantly reduced serum levels of TP, TG, GLU, and BUN, upregulated *tlr-2* and downregulated *tnf-α* expression, and modulated the gut microbiota by enriching *Agathobacter*, *Faecalibacterium* and *Anaerostipes* while decreasing *Acinetobacter* and *Pseudomonas*. The high-concentration HRW (550 ± 50 ppb, H2) significantly enhanced the activities of antioxidant enzymes SOD and CAT. No adverse effects on intestinal morphology or digestive enzymes were observed. These findings indicate that optimal HRW supplementation enhances immunomodulation, antioxidant defense, and intestinal health, positioning HRW as a promising functional additive for sustainable aquaculture. Further studies are needed to elucidate the underlying molecular mechanisms.

## Figures and Tables

**Table 1:** Primer sequence.

Gene	Accession Number	Primer Sequence	Tm (°C)	Product Size (bp)
*il-8*	NM_001310420.1	F:CTTCTCGGCTGTATCTGTG R:TTCCTCTTGCGACTCTTC	50.8	177
*tnf-a*	XM_067509868.1	F:ACAATACCACCCCAGGTCCCA R:ACGCAGCATCCTCTCATCCAT	61.8	251
*il-10*	XM_067517367.1	F:TGGCAGTGAAGAAGACAT R:CTTTGAAGTGCTCAGGGA	50.8	152
*hsp70*	XM_067518373.1	F:TTTCCAGGAGAGACTATGCG R:AACACCTTGGCCTGTTTAC	55.3	112
*tlr-2*	XM_067523858.1	F:TAAAGGAGGTGGAGACGT R:GAGGCGCAACAGCAAGAT	56.6	196
*tor*	XM_067527041.1	F:GAGCCTCTCTCATCCTCACCAC R:GATTCATTCCTTTCTCTTTAGCCA	60.1	129
*β-actin*	XM_067476706.1	F:CACTGTGCCCATCTACGAG R:CCATCTCCTGCTCGAAGTC	54.0	200

Note: the primer sequences were designed using Primer 6 software based on the gene sequences available in the NCBI database.

**Table 2:** Effects of HRW on growth performance of juvenile snakehead (*n* = 4).

Items	Groups		
	Control	H1	H2
Weight gain rate (WGR, %)	581.60 ± 19.73 ^ab^	635.41 ± 19.50 ^a^	566.74 ± 20.96 ^b^
Survival rate (SR, %)	99.17 ± 0.83	97.50 ± 0.83	99.17 ± 0.83
Feed rate (FR, %)	1.41 ± 0.01	1.36 ± 0.02	1.41 ± 0.02
Specific growth rate (SGR, %/d)	3.20 ± 0.03	3.28 ± 0.04	3.15 ± 0.07
Feed conversion ratio (FCR)	0.94 ± 0.01	0.90 ± 0.02	0.96 ± 0.02
Final weight (FW, g)	104.38 ± 3.01 ^ab^	112.70 ± 2.96 ^a^	102.13 ± 3.23 ^b^

Values are means + S.E. (*n* = 4). Different superscript letters in the same row indicate significant differences (*P* < 0.05).

**Table 3:** Effects of HRW on whole-body composition of juvenile snakehead (*n* = 4).

Items	Groups		
	Control	H1	H2
Crude protein (%)	18.80 ± 1.55	18.38 ± 0.41	18.88 ± 1.00
Crude fat (%)	6.58 ± 0.49	6.43 ± 0.69	6.72 ± 1.50
Moisture (%)	66.36 ± 1.03	67.61 ± 0.16	66.72 ± 0.55
Crude ash (%)	4.73 ± 0.51	4.64 ± 0.51	4.90 ± 0.74
Calcium (%)	1.43 ± 0.18	1.39 ± 0.17	1.47 ± 0.25
Phosphorus (%)	0.80 ± 0.08	0.79 ± 0.08	0.80 ± 0.14

Values are means + S.E. (*n* = 4). Differe the absence of superscript letters in the same row indicates no significant differences among groups (*P* > 0.05). All body composition values (crude protein, crude fat, crude ash, calcium, and phosphorus) are expressed as percentage of wet weight.

**Table 4:** Effect of HRW on serum biochemical indices in juvenile snakehead.

Items	Groups		
	Control	H1	H2
Total protein (TP, g/L)	46.80 ± 1.89 ^a^	38.58 ± 2.00 ^b^	43.28 ± 2.89 ^ab^
Triglyceride (TG, mmol/L)	1.25 ± 0.08 ^a^	0.95 ± 0.09 ^b^	1.04 ± 0.05 ^ab^
Total cholesterol (TC, mmol/L)	5.14 ± 0.28	4.37 ± 0.20	5.01 ± 0.35
High-density lipoprotein cholesterol (HDL-C, mmol/L)	3.46 ± 0.18	2.94 ± 0.11	3.28 ± 0.21
Low-density lipoprotein cholesterol (LDL-C, mmol/L)	0.54 ± 0.05	0.43 ± 0.05	0.55 ± 0.06
Glucose (GLU, mmol/L)	7.38 ± 0.32 ^a^	6.17 ± 0.47 ^b^	6.80 ± 0.30 ^ab^
Blood urea nitrogen (BUN, mmol/L)	1.04 ± 0.08 ^a^	0.70 ± 0.03 ^b^	0.84 ± 0.07 ^ab^

Values are means + S.E. (*n* = 4). Different superscript letters in the same row indicate significant differences (*P* < 0.05).

**Table 5:** Alpha diversity indices of microbial communities.

Items	Groups		
	Control	H1	H2
Ace	543.07 ± 113.43	486.74 ± 45.26	310.49 ± 96.75
Chao 1	540.13 ± 112.27	487.87 ± 44.43	307.33 ± 91.43
Shannon	2.63 ± 0.07	2.77 ± 0.40	1.41 ± 0.54
Simpson	0.16 ± 0.03 ^b^	0.21 ± 0.04 ^ab^	0.57 ± 0.18 ^a^
Sobs	498.00 ± 98.86	463.67 ± 44.76	273.67 ± 77.98

Values are means + S.E. (*n* = 3). Different superscript letters in the same row indicate significant differences (*P* < 0.05).

## Data Availability

All data generated or analyzed during this study are available from the corresponding authors upon reasonable request. The 16S rRNA gene sequencing data have been deposited in the GenBank database under accession number PRJNA1235928, and are accessible at https://www.ncbi.nlm.nih.gov/bioproject/PRJNA1235928 (accessed on 5 May 2026).
